# The SASP-associated long noncoding RNA *NEAT1* modulates the tumor immune microenvironment in hepatocellular carcinoma

**DOI:** 10.1016/j.iliver.2026.100277

**Published:** 2026-10-12

**Authors:** Yifei Wang, Runchen Miao, Sinan Liu, Rui Chen, Jie Ren, Kun Yang, Senyi Hu, Kaibo Yang, Kunjin Wu, Xiaohua Ma, Kai Qu, Chang Liu, Ting Lin

**Affiliations:** a Department of Surgical Critical Care, The First Affiliated Hospital of Xi'an Jiaotong University, Xi'an 710061, China; b Key Laboratory of Surgical Critical Care and Life Support (Xi'an Jiaotong University), Ministry of Education, Xi'an 710061, China; c Department of Hepatobiliary Surgery and Liver Transplantation, The Second Affiliated Hospital of Xi'an Jiaotong University, Xi'an 710004, China; d Department of Biosciences, University of Liverpool, Liverpool L69 7ZX, United Kingdom

**Keywords:** Senescence-associated secretory phenotype, Hepatocellular carcinoma, Long noncoding RNA, *NEAT1*, Tumor immune microenvironment

## Abstract

**Background and aims:**

Cellular senescence exerts dual effects on tumorigenesis: it can prevent malignant transformation, and the senescence-associated secretory phenotype (SASP) can remodel the tumor microenvironment and influence cancer progression. Long noncoding RNAs (lncRNAs) have emerged as important SASP regulators; however, their roles in hepatocellular carcinoma (HCC) remain poorly understood.

**Methods:**

We performed an integrated analysis of Gene Expression Omnibus datasets to identify SASP-associated lncRNAs in HCC and validated the candidates in hydrogen peroxide–induced senescent HCC cell lines (HepG2 and Huh7). Western blotting, immunofluorescence, and enzyme-linked immunosorbent assay were used to investigate the regulatory mechanism. TIMER database and survival analyses were used to determine associations with the immune microenvironment.

**Results:**

*NEAT1* and *DLEU2* were identified as core SASP-associated lncRNAs, and *NEAT1* showed strong positive correlation with the SASP factors such as interleukin (IL)-6, IL-8, and IL-1β. *NEAT1* knockdown or NF-κB pharmacological inhibition significantly reduced H_2_O_2_-induced SASP secretion by suppressing p65 phosphorylation and nuclear translocation, indicating that *NEAT1* promotes SASP production by activating the NF-κB pathway. Furthermore, *NEAT1* expression was positively correlated with infiltration of CD8^+^ cells, CD4^+^ T cells, and macrophages in HCC tissues, and elevated *NEAT1* expression was associated with favorable prognosis in patients with HCC.

**Conclusion:**

We identified *NEAT1* as a novel SASP-associated lncRNA that may shape an antitumor immune microenvironment in HCC through NF-κB-dependent SASP regulation. These findings indicate that *NEAT1* may be a key prognostic biomarker and therapeutic target in HCC.

## 1 Introduction

Liver cancer is among the commonly diagnosed malignancies and the third leading cause of cancer-related mortality worldwide, accounting for approximately 830,000 deaths.^1^ China is among the countries at highest risk, and hepatocellular carcinoma (HCC) is the most prevalent primary liver cancer, with chronic hepatitis B virus infection and aflatoxin exposure being the key determinants.^2^ Approximately 80% of cases are diagnosed in patients aged ≥70 years and at an advanced stage, limiting treatment efficacy.^3^ Therefore, a better understanding of liver cancer pathogenesis is crucial for successful treatment.

HCC is closely associated with persistent chronic inflammation,^4^ which is also a key driver of aging and age-related diseases.^5^, ^6^, ^7^ Because cellular senescence is a hallmark of aging, it may contribute substantially to HCC development and progression.^8^, ^9^ Cellular senescence is a state of stable growth arrest with continued metabolic activity in response to various forms of damage and stress. Senescent cells can develop a distinct senescence-associated secretory phenotype (SASP) and secrete proinflammatory cytokines, growth factors, chemokines, and metalloproteases. These factors affect neighboring tissues by attracting immune cells, promoting angiogenesis, and remodeling the extracellular matrix.^10^, ^11^ Variations in SASP composition may determine the contrasting effects of senescent cells in different organs.^12^ In premalignant stages, senescent liver cancer cells allow immune cell recruitment to lesion sites, preventing the proliferation of damaged cells and reducing carcinogenic risk. However, in chronic liver diseases, abnormal senescent liver cells may alter the microenvironment by releasing large quantities of proinflammatory cytokines, chemokines, and proteases.^13^ SASP can regulate hepatic immune responses and sustain a proinflammatory environment that may damage cellular components including organelles and DNA.^14^, ^15^ Thus, SASP is a key mediator of the harmful effects of senescent cells and contributes to tumor development and progression including that of HCC.

Although SASP regulation has been reported to occur at several levels including chromatin remodeling and post-transcriptional control, most SASP components are regulated at the transcriptional level.^16^ Two principal transcription factors enable SASP activation in response to senescence-inducing stimuli: C/EBP and NF-κB.^17^, ^18^ Long noncoding RNAs (lncRNAs) are transcripts longer than 200 nucleotides that generally lack protein-coding capacity and regulate gene expression through several processes including transcription.^19^, ^20^, ^21^ They participate in numerous cellular processes including proliferation, senescence, apoptosis, and immune responses.^22^ Accumulating evidence links altered lncRNA expression to proinflammatory phenotypes in age-related diseases.^23^, ^24^ Therefore, lncRNAs may be important SASP regulators, warranting the identification of additional SASP-associated lncRNAs.

In this study, we aimed to characterize SASP secretion by senescent HCC cells, identify lncRNAs that may regulate these factors, and examine the relationships between these lncRNAs and the HCC immune microenvironment. The broader aim of this study was to identify potential therapeutic strategies for liver cancer based on the regulation of cellular senescence.

## 2 Materials and methods

### 2.1 Data acquisition and processing

We obtained raw datasets for IMR-90 senescence models from the Gene Expression Omnibus (GEO) database (http://www.ncbi.nlm.nih.gov/geo/). All datasets were generated using the Affymetrix HG-U133 Plus 2.0 gene-chip microarray platform. IMR-90 cells were selected as the source of senescence profiles because they are widely used to model replicative senescence and because the four datasets were generated on the same microarray platform with matched proliferating controls. This screen was used solely to identify candidates, and all selected lncRNAs and cytokines were subsequently validated in HCC cell lines. After normalization and annotation, the GSE19018, GSE19864, GSE36640, and GSE60652 gene expression profiles were examined using gene set enrichment analysis (GSEA) to identify pathways differentially enriched between senescent and proliferating groups.

### 2.2 Enrichment analysis

GSEA software (version 4.1.0, Broad Institute, Cambridge, MA, USA) was used to evaluate the distribution of senescence-related genes across the senescent groups. BIOCARTA and Kyoto Encyclopedia of Genes and Genomes (KEGG) pathway analyses were performed to identify inflammation-related pathways enriched in senescent or proliferating profiles, using a false discovery rate of <0.05. A total of 1000 random permutations were performed.

### 2.3 Identification of differentially expressed genes between the senescent and proliferating groups

The DESeq2 and limma packages in R were used to identify senescence-related differentially expressed genes. Differential expression was defined using |log_2_ fold change| >1 and −log_10_ *p* > 1.3 as the screening thresholds.

### 2.4 In vitro senescence induction in HCC cells

HepG2 cells were obtained from the Shanghai Institute of Biochemistry and Cell Biology, Chinese Academy of Sciences (Shanghai, China), and Huh7 cells were provided by Shanghai Fuxiang Biotechnology (Shanghai, China). Human HCC cell lines were cultured in Dulbecco's modified Eagle's medium (HyClone, GE Healthcare Life Sciences, Logan, UT, USA) supplemented with 10% fetal bovine serum (Gibco, Thermo Fisher Scientific, Inc., Waltham, MA, USA) and 1% V/V penicillin/streptomycin (MilliporeSigma, Bedford, MA, USA). Cells were incubated at 37 °C in a 95% humidified atmosphere containing 5% CO_2_.

Cells were treated with 100 μM H_2_O_2_ (VWR Inc., West Chester, PA, USA) in complete medium for 24 h to induce senescence. This regimen is consistent with published protocols in which H_2_O_2_ induced senescence-associated β-galactosidase positivity in HepG2 cells.^25^ Control cells were cultured in parallel without H_2_O_2_. The senescent state was operationally defined by the secretion of interleukin (IL)-1α, IL-1β, IL-6, and IL-8, which are recognized components of the SASP.^26^ To investigate the function of the NF-κB pathway in *NEAT1*-mediated SASP regulation, Huh7 cells were cotreated for 24 h with 100 μM H_2_O_2_ and 10 μM BAY11-7082 (HY-13453, MedChemExpress, Monmouth Junction, NJ, USA), a specific NF-κB inhibitor. For *NEAT1* knockdown, cells were transfected with si-*NEAT1* or negative control siRNA using Lipofectamine 2000 (Invitrogen, Carlsbad, CA, USA) before H_2_O_2_ treatment.

### 2.5 Cell viability assay

HepG2 and Huh7 cells were seeded in 96-well plates at a density of 4 × 10^3^ cells/well. After attachment, the cells were treated with 100 μM H_2_O_2_ for 24 h or left untreated. At 0, 24, and 48 h, 10 μL of CCK-8 reagent (Mishu Biotechnology, Xi'an, China) was added to each well, and the plates were incubated at 37 °C for 2 h. Absorbance was measured at 450 nm. Each assay was performed in six replicate wells.

### 2.6 RNA extraction and real-time quantitative polymerase chain reaction (PCR)

Cytokine production in HCC cells after senescence induction was assessed by RNA extraction, reverse transcription, and RT-PCR. Total cellular RNA was isolated using TRIzol (Invitrogen, Carlsbad, CA, USA), and cDNA was synthesized from mRNA using cDNA synthesis kits (Fermentas, USA). qRT–PCR was performed using SYBR Green dye (TaKaRa Bio Inc., Otsu, Japan). All assays were conducted in triplicate according to the manufacturers’ protocols. The mRNA expression was normalized to that of glyceraldehyde-3-phosphate dehydrogenase (*GAPDH*) and calculated using the comparative 2^−ΔΔCt^ method.

We used the following primer pairs for qRT–PCR: *GAPDH*, forward, 5′-GTCTCCTCTGACTTCAACAGCG-3′; reverse, 5′-ACCACCCTGTTGCTGTAGCCAA-3′. IL-1α, forward, 5′-TGGTAGTAGCAACCAACGGGA-3′; reverse, 5′-ACTTTGATTGAGGGCGTCATTC-3′. IL-1β, forward, 5′-ACTTGGGCTGTCCAGATGAG-3′; reverse, 5′-GTAGCTGCCACAGCTTCTCC-3′. IL-6, forward, 5′-ACTGGTTCTGTGCCTGCAGC-3′; reverse, 5′-CAGGAGAAGATTCCAAAGAT-3′. IL-8, forward, 5′-CACAAGAGCCAGGAAGAAAC-3′; reverse, 5′-CTACAACAGACCCACACAATAC-3′. All primer pairs were synthesized by TaKaRa Bio Inc. (Otsu, Japan).

### 2.7 Western blotting

Cells were harvested and lysed in RIPA buffer (Beyotime Biotechnology, Shanghai, China), and the lysates were centrifuged at 12,000 rpm for 20 min at 4 °C. The supernatants were collected, and equal amounts of protein were separated by sodium dodecyl sulfate–polyacrylamide gel electrophoresis and transferred to nitrocellulose membranes (MilliporeSigma, Bedford, MA, USA). The membranes were blocked with 4% skim milk in phosphate-buffered saline with Tween 20 (PBST) for 2 h at room temperature and incubated overnight at 4 °C with primary antibodies against p65 (1:5000; YM8209; Immunoway, Plano, TX, USA), p-p65 (1:2000; YM8442; Immunoway, Plano, TX, USA), and GAPDH (1:1000; GB15002-100; Servicebio, Wuhan, China). After three washes with PBST, the membranes were incubated for 1 h at room temperature with horseradish peroxidase–conjugated secondary antibodies (goat anti-rabbit IgG, 1:1000, E-AB-1003, Elabscience, Wuhan, China; goat anti-mouse IgG, 1:1000, E-AB-1001, Elabscience, Wuhan, China). Protein bands were visualized using enhanced chemiluminescence substrate (Beyotime Biotechnology, Shanghai, China), and the images were analyzed using ImageJ version 1.53 (version 1.53; National Institutes of Health, Bethesda, MD, USA).

### 2.8 Immunofluorescence

Huh7 cells were seeded onto glass coverslips in 24-well plates and subjected to the indicated treatments. Subsequently, the cells were washed with phosphate-buffered saline (PBS, C0221A; Beyotime Biotechnology, Shanghai, China), fixed with 4% paraformaldehyde (P0099; Beyotime Biotechnology, Shanghai, China) for 15 min at room temperature, and permeabilized with 0.3% Triton X-100 (ST795; Beyotime Biotechnology, Shanghai, China) for 10 min. After blocking with 5% bovine serum albumin (ST023; Beyotime Biotechnology, Shanghai, China) in PBS for 1 h at room temperature, the cells were incubated overnight at 4 °C with a primary antibody against p65 (1:200; YM8209; Immunoway, Plano, TX, USA). After three washes with PBS, the cells were incubated in the dark for 1 h at room temperature with a fluorescein isothiocyanate–conjugated secondary antibody (1:500; Servicebio, Wuhan, China). Nuclei were counterstained with DAPI (1 μg/mL; Sigma-Aldrich, Sigma-Aldrich, St. Louis, MO, USA) for 5 min. Fluorescence images were acquired using a fluorescence microscope (Olympus, Tokyo, Japan). Nuclear p65 fluorescence intensity was quantified in at least three randomly selected fields per group using ImageJ.

### 2.9 Enzyme-linked immunosorbent assay (ELISA)

The concentrations of cytokines released into the culture medium after H_2_O_2_-induced senescence were measured by ELISA. All ELISA kits were purchased from Enzo Life Sciences (Farmingdale, NY, USA). The standards and samples were added according to the manufacturer's instructions. IL-1α, IL-1β, IL-6, and IL-8 levels were measured at 450 nm using a Shimadzu spectrophotometer (Shimadzu Corporation, Kyoto, Japan) and normalized to the absorbance of the standard solution. All assays were performed in three independent experiments.

### 2.10 Construction of weighted gene coexpression network

After differential expression analysis identified differentially expressed (DE)-mRNAs and DE-lncRNAs, module membership was assessed using the WGCNA package in R. A topological overlap matrix was used to assess gene-network connectivity, and average-linkage hierarchical clustering was used to identify gene coexpression modules. The modules were assigned arbitrary colors to facilitate correlation analysis, and those with high correlation coefficients were selected as candidates. Next, we identified DE-lncRNAs closely associated with IL-1β, IL-6, or IL-8. The intersection of lncRNAs associated with these cytokines in senescent cells was visualized using the VennDiagram R package. Pearson's test was used to confirm correlations between overlapping lncRNAs and IL-1β, IL-6, or IL-8.

### 2.11 RNA interference and plasmid transfection

Small interfering RNAs (siRNAs) targeting *NEAT1* and a negative control were synthesized by Shanghai Gene Pharma Co. (Shanghai, China). At 70% confluence, the cells were transfected with siRNA-negative control (NC), si-*NEAT1*#1, or si-*NEAT1*#2 using Lipofectamine 2000 (Invitrogen, Carlsbad, CA, USA). The siRNA sequences of lncRNA *NEAT1* were as follows: si-*NEAT1*#1, forward, 5′-GUGAGAAGUUGCUUAGAAATT-3′ and reverse, 5′-UUUCUAAGCAACUUCUCACTT-3′; si-*NEAT1*#2, forward, 5′-AGCTTCCAAAAAAGGGTTCGTTGAGAGCTCAAAGCTA-3′ and reverse, 5′-ATCTCTTGAATTAGCTTTGAGCTCTCAACGAACGCCGA-3′. The scrambled NC sequences were as follows: forward, 5′-GATCTCGGACTCGCGGTTTGTTGTGATTCTCTTTCAAGA-3′ and reverse, 5′-GAAGAGAATCACAACAAACCGCGAGTCCTTTTTTGGA-3′.

The pcDNA3.1-*DLEU2* overexpression plasmid was generated by subcloning PCR-amplified full-length human *DLEU2* cDNA into the pcDNA3.1 vector (Thermo Fisher Scientific, Inc. Waltham, MA, USA). Equal amounts of empty pcDNA3.1 vector were used as the control.

### 2.12 KEGG analysis

After 459 *NEAT1*-coexpressed DE genes were identified by weighted gene coexpression network analysis (WGCNA), KEGG pathway-enrichment analysis was performed using the ClusterProfiler R package to investigate their potential biological functions and underlying mechanisms.

### 2.13 Immune infiltration analysis

The association between *NEAT1* and immune cell infiltration in HCC was examined using Tumor Immune Estimation Resource (TIMER) 2.0 (http://timer.cistrome.org/), a comprehensive web-based tool for immune infiltration analysis. We focused on the relationships between *NEAT1* copy number subgroups and the abundance of tumor-infiltrating neutrophils, B cells, CD8^+^ T cells, CD4^+^ T cells, macrophages, and dendritic cells.

### 2.14 Statistical analyses

Experimental data are presented as means ± standard deviation and were analyzed using GraphPad Prism version 9.0 (GraphPad Inc., San Diego, CA, USA). For immunofluorescence quantification, nuclear p65 fluorescence intensity was measured in at least three randomly selected fields per group using ImageJ version 1.53 (National Institutes of Health, Bethesda, MD, USA). The mean of the three fields was calculated for each independent experiment. The two groups were compared using the unpaired two-tailed Student's *t*-test. R Studio (Posit, Boston, MA, USA) was used to run all aforementioned R packages. Correlations between the expression of two genes were assessed using Spearman correlation analysis.

## 3 Results

### 3.1 Identification of inflammatory pathways enriched in senescence-related genes

Gene expression profiles from 14 senescent and 14 proliferating IMR-90 cell samples in GSE19018, GSE19864, GSE36640, and GSE60652 were normalized and annotated (Fig. 1A). GSEA was used to identify key inflammatory pathways that differed between the senescent and proliferating groups. Three core inflammatory pathways were identified (Fig. 1B–D). BIOCARTA analysis indicated enrichment of inflammation-related genes, whereas KEGG analysis indicated enrichment of cytokine–cytokine receptor interaction and chemokine signaling pathways.

**Fig. 1:**
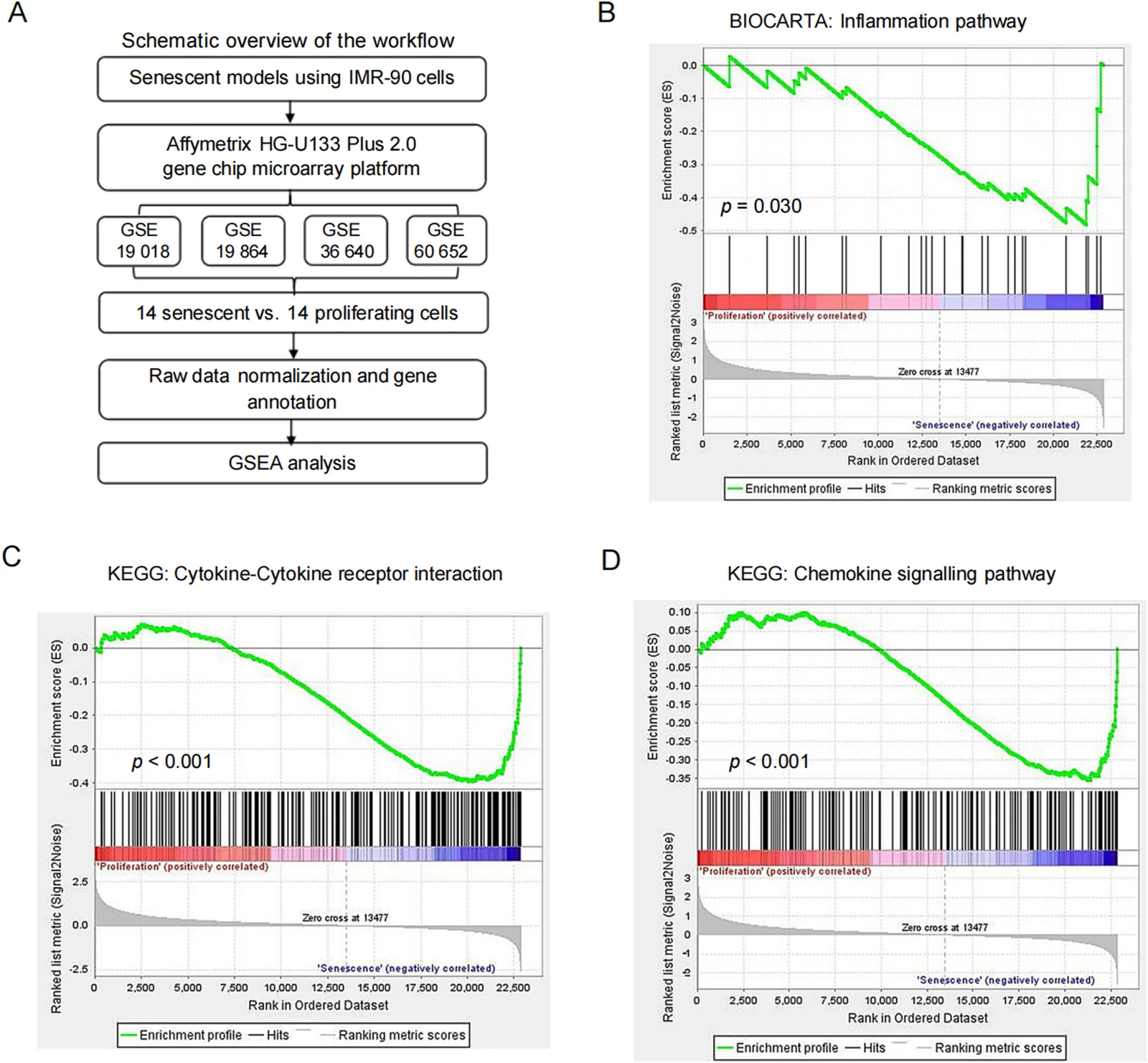
Gene set enrichment analysis (GSEA) of senescence-related genes. (A) Flowchart of data acquisition and processing. (B–D) GSEA enrichment plots of the core inflammatory pathways in senescence-related genes: BIOCARTA inflammation pathway (B), Kyoto Encyclopedia of Genes and Genomes (KEGG) cytokine-cytokine receptor interaction (C), and KEGG chemokine signalling pathway (D).

### 3.2 Verification of cytokine release from senescent HCC cells in vitro

Considering the essential roles of cytokines in these inflammatory pathways, we examined cytokines differentially expressed between senescent and proliferating cells in the datasets. The top four differentially expressed cytokines were IL-8, IL-1β, IL-1α, and IL-6 (Fig. 2A). Briefly, HCC cells were treated with 100 μM H_2_O_2_ for 24 h to induce senescence; the culture supernatants and cells were then collected to measure cytokine release and expression, respectively (Fig. 2B). Next, we validated these findings in HepG2 and Huh7 HCC cells in which senescence had been induced using H_2_O_2_ (Fig. 2C and D). Expression of IL-8, IL-1β, IL-1α, and IL-6 was higher in senescent HCC cells than in proliferating cells. Senescent HCC cells also released more of these four cytokines, confirming the presence of a SASP in senescent HCC cells. In both cell lines, CCK-8 assays showed that H_2_O_2_-treated cells remained metabolically active: viability plateaued rather than declining during the 48-h observation period, whereas control cells continued to proliferate (Fig. 2E and F). No substantial cell death was observed.

**Fig. 2:**
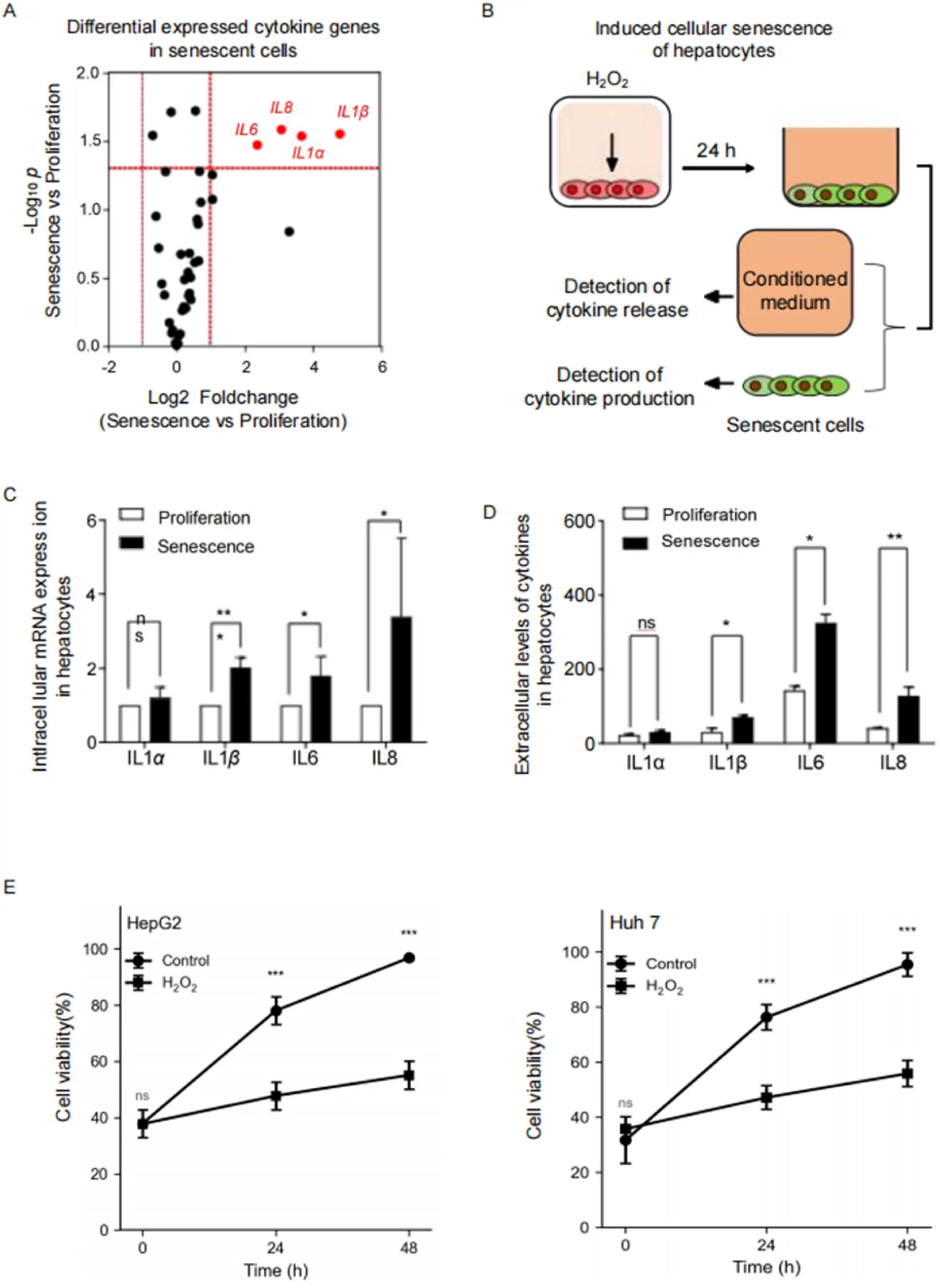
Validation of cytokines differentially expressed in senescent HepG2 and Huh7 hepatocellular carcinoma (HCC) cells. (A) Differential analysis of cytokines in senescence-related Gene Expression Omnibus (GEO) datasets. (B) Procedure used to induce senescence in HCC cells. (C) Cytokine expression in senescent HCC cells assessed by quantitative reverse transcription polymerase chain reaction (qRT–PCR). (D) Cytokine levels in culture media from senescent HCC cells assessed by enzyme-linked immunosorbent assay (ELISA). (E) Cell viability of HepG2 (left) and Huh7 (right) cells measured by CCK-8 assay at the indicated time points after treatment with or without 100 μM H₂O₂ for 24 h (*n* = 6). ∗*p* < 0.05, ∗∗*p* < 0.01, ∗∗∗*p* < 0.001, ns, not significant.

### 3.3 Acquisition of vital lncRNAs associated with SASP released by senescent HCC cells

We first performed WGCNA to identify lncRNAs closely associated with the core cytokines. This analysis identified a module of 1130 lncRNAs coexpressed with IL-8, IL-1β, and IL-6 (Fig. 3A). These 1130 cytokine-associated lncRNAs were intersected with the lncRNAs differentially expressed between senescent and proliferating cells (Fig. 3B), followed by correlation analysis. Ultimately, two core lncRNAs, *NEAT1* and *DLEU2*, were identified. *NEAT1* was positively correlated with the cytokines, whereas *DLEU2* was negatively correlated with them (Fig. 3C). These relationships were subsequently validated in the four initial GEO datasets (Fig. 4A–C).

**Fig. 3:**
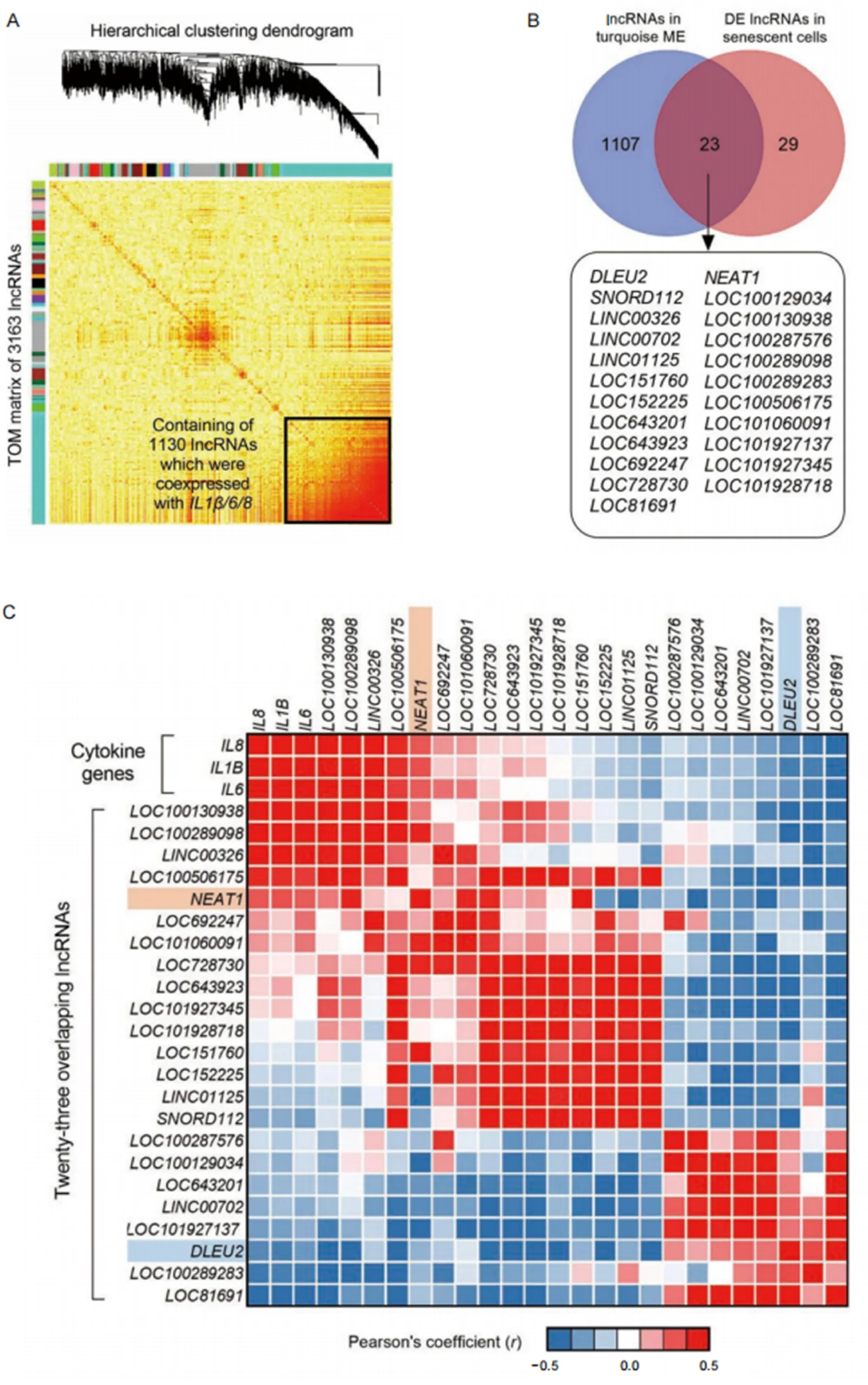
Identification of senescence-associated secretory phenotype (SASP)-coexpressed long noncoding (lnc) RNAs. (A) LncRNA modules coexpressed with interleukin (IL)-1β, IL-6, and IL-8 identified by weighted gene coexpression network analysis (WGCNA). (B) Intersection between cytokine-associated lncRNAs and lncRNAs differentially expressed in the datasets. (C) Correlations between the intersecting lncRNAs and IL-1β, IL-6, and IL-8. DE, differentially expressed; ME, module eigengene; TOM, topological overlap matrix.

**Fig. 4:**
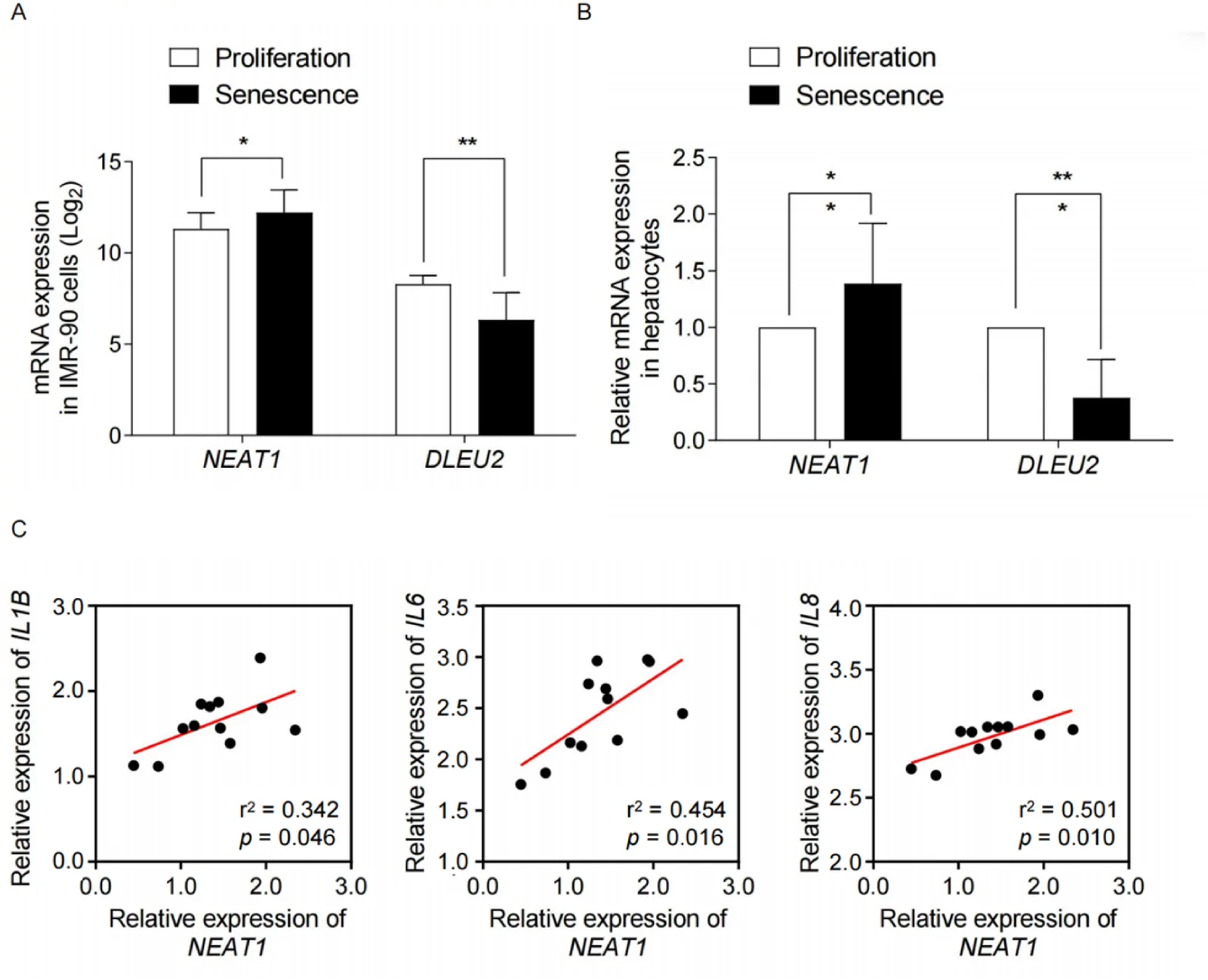
Validation of key long noncoding (lnc) RNA expression in senescent cells and correlations with cytokines. (A) *NEAT1* and *DLEU2* mRNA expression in senescent and proliferating IMR-90 cells. (B) *NEAT1* and *DLEU2* mRNA expression in senescent and proliferating hepatocytes. (C) Correlations between *NEAT1* expression and interleukin (IL)-1β, IL-6, and IL-8 expression.

### 3.4 Verification of the regulatory role of vital lncRNAs in SASP

The aforementioned dataset analyses established associations between *NEAT1* and *DLEU2* and the major SASP factors IL-8, IL-1β, and IL-6. Next, we generated *NEAT1*-knockdown (si-*NEAT1*) and *DLEU2*-overexpressing HCC cells and validated the altered expression using qRT–PCR (Fig. 5A and B). After selecting stably expressing HepG2 and Huh7 cell lines, we used ELISA to compare SASP levels in proliferating HCC cells, senescent HCC cells, and senescent HCC cells with altered regulatory lncRNA expression. In both cell lines, reduced *NEAT1* expression reversed the increased release of IL-8, IL-1β, and IL-6 from senescent HCC cells (Fig. 5C–F). *DLEU2* overexpression reduced SASP release by senescent HCC cells. Thus, *NEAT1* positively regulated SASP release, whereas DLEU2 regulated it negatively.

**Fig. 5:**
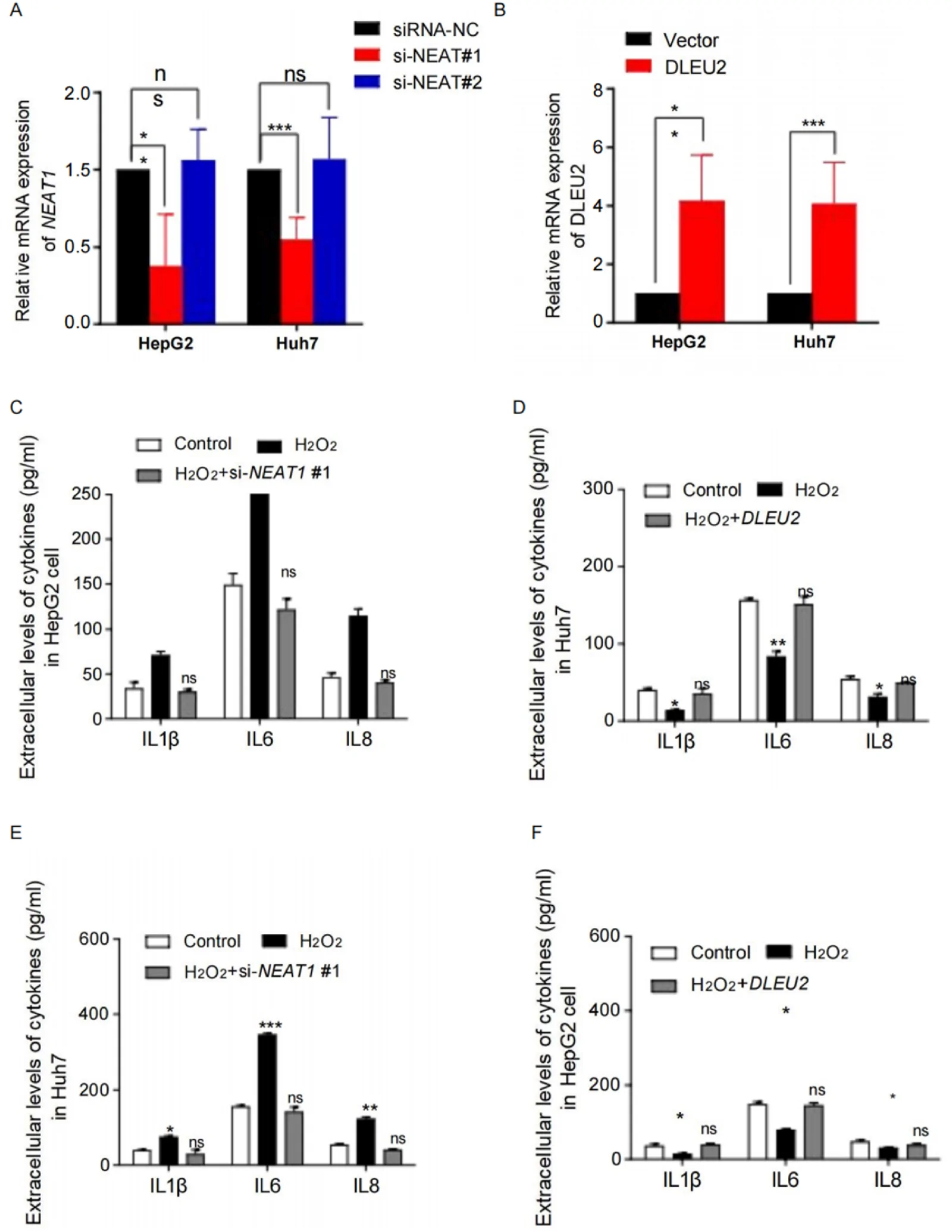
Extracellular cytokine levels after *NEAT1* knockdown or *DLEU2* overexpression. (A, B) Construct selection and expression validation after *NEAT1* knockdown and *DLEU2* overexpression, respectively. (C, E) Extracellular interleukin (IL)-1β, IL-6, and IL-8 levels after *NEAT1* knockdown in HepG2 and Huh7 cells, respectively. (D, F) Extracellular IL-1β, IL-6, and IL-8 levels after *DLEU2* overexpression in HepG2 and Huh7 cells, respectively. ∗*p* < 0.05, ∗∗*p* < 0.01, ∗∗∗*p* < 0.001.

### 3.5 Identification of NF-κB as a key mediator of *NEAT1*-regulated SASP

Our preliminary experiments showed that *NEAT1* expression was positively correlated with secretion of the SASP factors IL-6, IL-8, and IL-1β and that *NEAT1* knockdown significantly suppressed their secretion in senescent HCC cells. The NF-κB signaling pathway is a central transcriptional regulator of inflammatory factor expression and has been extensively implicated in SASP secretion. To elucidate the specific molecular mechanism by which *NEAT1* regulates SASP, we examined the phosphorylation and nuclear translocation of p65, a key NF-κB subunit.

Pathway validation was performed in Huh7 cells. Western blotting was used to measure p65 phosphorylation in four treatment groups. Compared with the control group, H_2_O_2_ treatment significantly increased p-p65 expression (*p* < 0.001), indicating NF-κB pathway activation after senescence induction (Fig. 6A and B). *NEAT1* knockdown markedly reversed the H_2_O_2_-induced increase in p-p65 (*p* < 0.01), producing an inhibitory effect comparable to that of the specific NF-κB inhibitor BAY11-7082. We assessed p65 subcellular localization by immunofluorescence. In control cells, p65 was predominantly cytoplasmic, with weak nuclear fluorescence (Fig. 6C and D). H_2_O_2_ treatment markedly increased nuclear green fluorescence, whereas *NEAT1* knockdown or BAY11-7082 treatment significantly suppressed p65 nuclear translocation and reduced nuclear fluorescence intensity to levels comparable to those in the control group. Finally, ELISA showed that H_2_O_2_-induced senescence significantly increased IL-1β, IL-6, and IL-8 secretion (Fig. 6E). *NEAT1* knockdown markedly reversed these increases, with effects similar to those of BAY11-7082. Collectively, these findings indicate that the NF-κB pathway is a critical downstream effector of *NEAT1*-mediated SASP regulation.

**Fig. 6:**
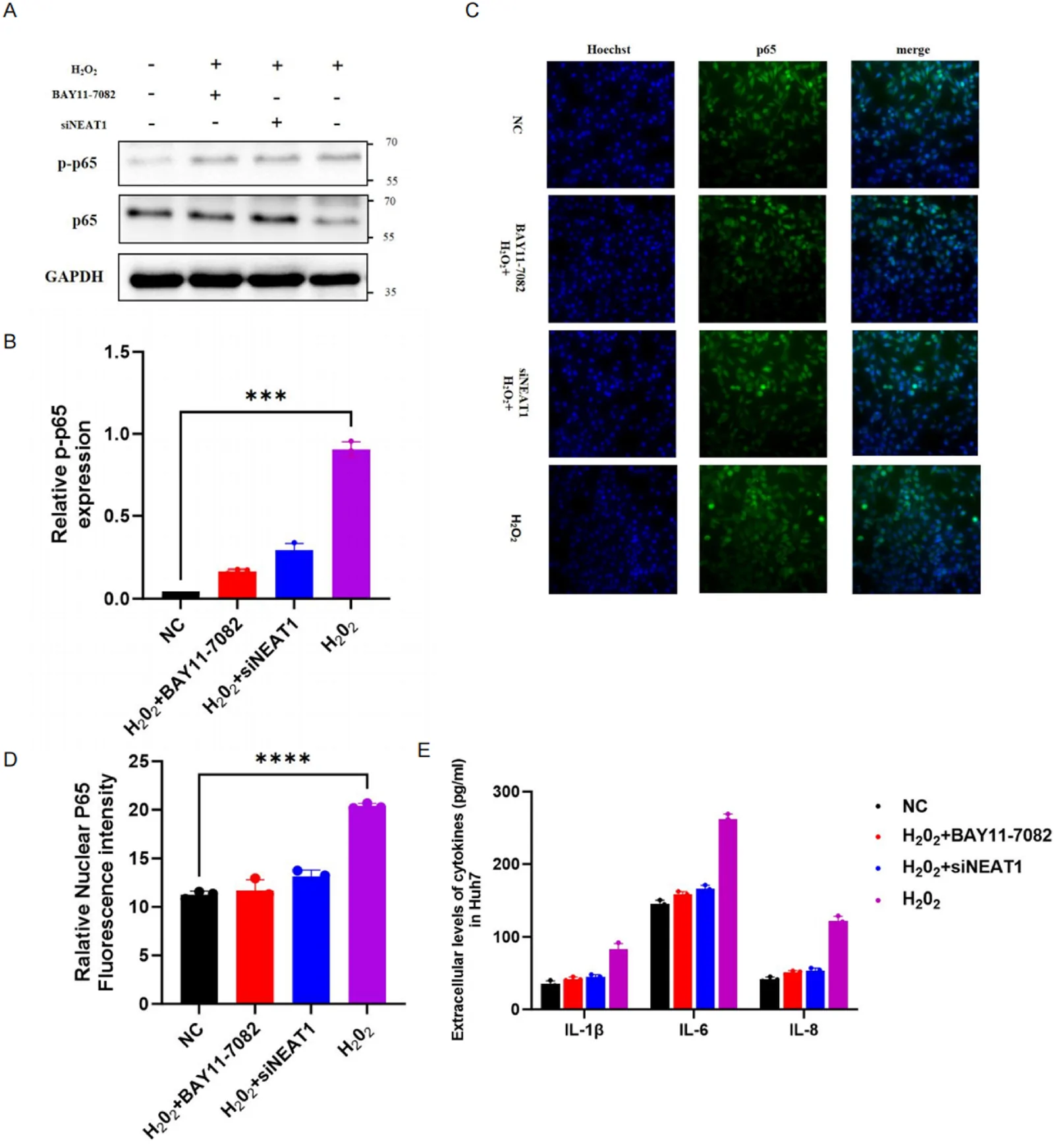
*NEAT1* promotes senescence-associated secretory phenotype (SASP) expression by activating the NF-κB pathway. (A, B) Western blot analysis and quantification of p65 and p-p65 expression (*n* = 3). Huh7 cells were assigned to four treatment groups: control, H_2_O_2_-induced senescence, H_2_O_2_ plus *NEAT1* knockdown (H_2_O_2_ + si-*NEAT1*), and H_2_O_2_ plus the specific NF-κB inhibitor BAY11-7082 (H_2_O_2_ + BAY11-7082). (C, D) Immunofluorescence analysis of p65 in Huh7 cells, with DAPI nuclear counterstaining (*n* = 3). (E) Enzyme-linked immunosorbent assay of the core SASP factors interleukin (IL)-6, IL-8, and IL-1β in Huh7 cells. ∗∗∗*p* < 0.001, ∗∗∗∗*p* < 0.0001.

### 3.6 Exploration of the pathways and immune infiltration of *NEAT1*-related senescence genes

Considering the close association between *NEAT1* and the SASP, we performed WGCNA and KEGG pathway-enrichment analyses of senescence-related genes coexpressed with *NEAT1*. We identified 459 such genes; the top three enriched pathways included those in cancer, cytokine–cytokine receptor interaction, and human T-cell leukemia virus type 1 (HTLV-1) infection (Fig. 7A and B). Altered *NEAT1* copy number was also correlated with the infiltration of neutrophils, CD8^+^ T cells, CD4^+^ T cells, and macrophages in HCC (Fig. 7C and D). These findings suggest that *NEAT1* influences HCC development by modulating SASP levels and altering antitumor immunity. Finally, The Cancer Genome Atlas (TCGA) liver cancer data analysis showed that higher *NEAT1* expression was associated with better survival (*p* = 0.018) (Fig. 7E).

**Fig. 7:**
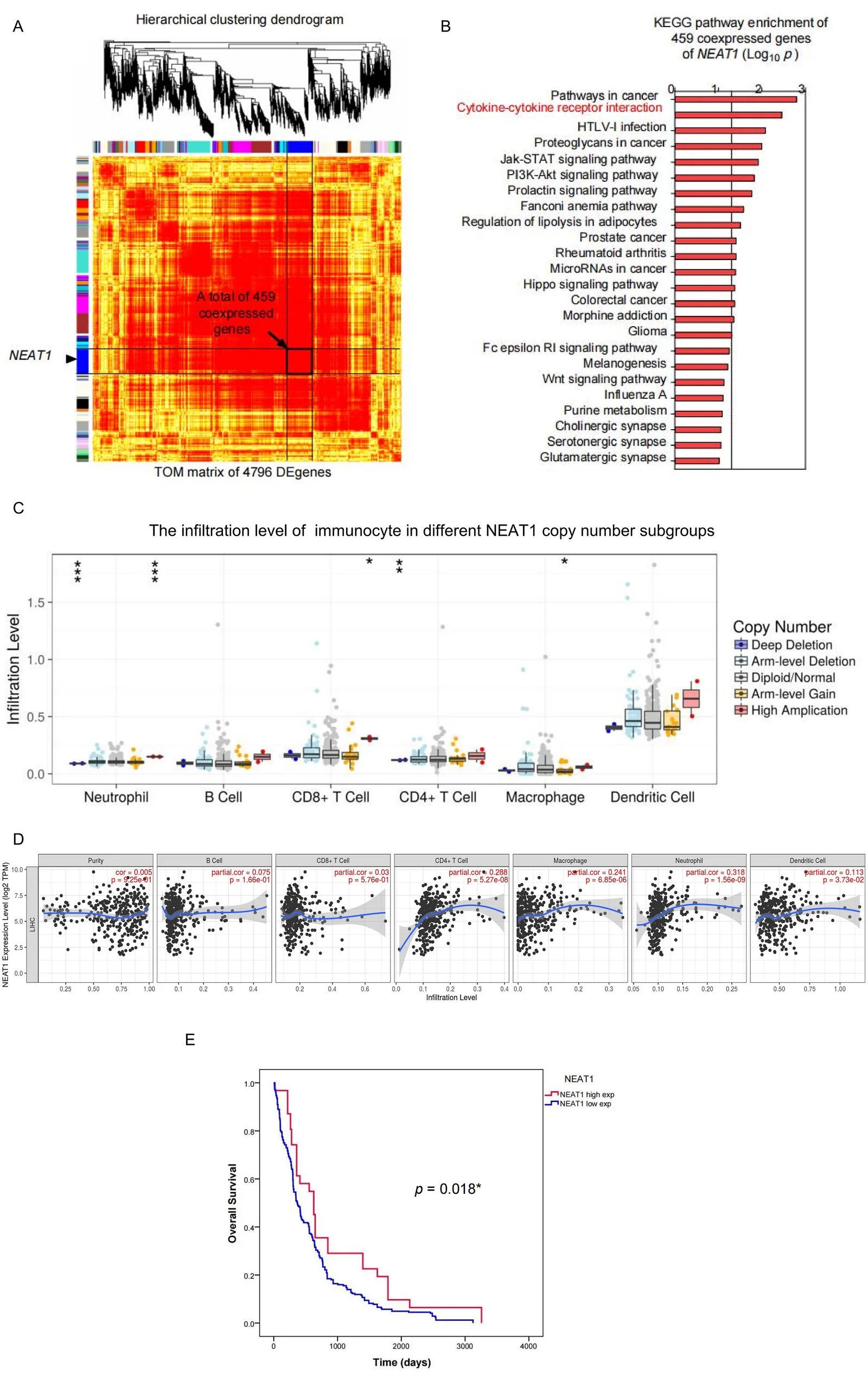
Role of *NEAT1* in hepatocellular carcinoma (HCC). (A) Weighted gene coexpression network analysis (WGCNA) of senescence-related differentially expressed genes coexpressed with *NEAT1*. (B) Kyoto Encyclopedia of Genes and Genomes analysis of *NEAT1*coexpressed genes. (C) Relationship between *NEAT1* copy number variation and immune cell infiltration in HCC. (D) Correlation between *NEAT1* and immune infiltration in HCC. (E) Kaplan–Meier survival curve for *NEAT1* in HCC. ∗*p* < 0.05, ∗∗*p* < 0.01, ∗∗∗*p* < 0.001.

## 4 Discussion

The prognosis of liver cancer is extremely poor. Very few patients with early-stage disease are eligible for surgical resection, and most patients present with advanced disease and undergo ablation, chemotherapy, or immunotherapy. The long-term outcomes of these treatments remain unsatisfactory, highlighting the need for novel therapeutic approaches. Regulation of senescent cells and SASP has emerged as a potential treatment strategy for several tumors. Considering the dual role of SASP in cancer, new therapies aim to preserve its beneficial effects while suppressing its harmful effects.^27^ To identify potential intervention targets in HCC, we characterized SASP secretion by senescent HCC cells, identified core SASP-associated lncRNAs, and examined their associations with the immune microenvironment and prognosis.

In vivo senescent cell accumulation primarily depends on SASP secretion for its role in promoting chronic inflammation and interfering with the immune microenvironment of cancer.^13^ However, the specific function and mechanism of SASPs in the surrounding tissues and immune microenvironment may differ according to cell type and physiological context. Schnabl B et al. reported that activated human hepatic stellate cells (HSCs) shift from a fibrogenic to a more inflammatory phenotype during aging in culture, thereby modulating chronic wound-healing processes.^28^ Another study reported that factors secreted by activated human HSCs may contribute directly to liver fibrosis.^29^ Activated HSCs also exhibit an aging-induced proinflammatory shift in the expression of major SASP components IL-6, Gro-α, and CXCL9, which promote obesity-associated HCC development in mice.^30^ Therefore, the effects of SASP in tumors depend on the senescent cell type and disease stage. At advanced stages, SASP factors released by senescent cancer-associated fibroblasts can stimulate cancer cell proliferation and tumor progression.^13^ In this study, integration of four aging-related GEO datasets confirmed a close association between aging and inflammation. Subsequently, we identified four core cytokines (IL-8, IL-1β, IL-1α, and IL-6) and validated their expression and release in H_2_O_2_-induced senescent HCC cells and their culture media. These findings indicate that senescent HCC cells release SASP factors that may influence the tumor immune microenvironment through autocrine and paracrine mechanisms.

The screening approach had an important limitation: the discovery datasets were derived from IMR-90 lung fibroblasts rather than hepatocytes. We accepted this trade-off because senescence microarray series from liver cells are limited in public databases, whereas the IMR-90 series provided four independent, platform-matched comparisons with adequate sample sizes. The cytokines selected through this screen are among the SASP factors most consistently induced across cell types and species. Moreover, their induction and their correlations with *NEAT1* were reproduced experimentally in two HCC cell lines.

Early studies have established important roles for lncRNAs in inflammatory regulation, prompting further investigation of their complexity and importance in SASP control. Peng et al. showed that lncRNA *PANDA* overexpression promoted HCC proliferation and carcinogenesis in vitro and in vivo by repressing transcription of the senescence-associated inflammatory factor IL-8, an effect associated with inhibition of cellular senescence.^31^ Silencing lncRNA-*LET*, which is expressed at low levels in tumors, including HCC, was proposed to promote nuclear factor 90 (NF90) accumulation in senescent cells, thereby reducing production of SASP factors such as CCL2, CXCL1, and IL-6.^32^, ^33^ To identify lncRNAs that regulate the core cytokines expressed at high levels in senescent HCC cells, we performed WGCNA and identified several lncRNAs coexpressed with these cytokines. Subsequent correlation analyses identified *NEAT1* and *DLEU2* as two key lncRNAs for validation. *NEAT1* has been shown to facilitate IL-8 transcription by relocating SFPQ to nuclear paraspeckles after immune stimulation, consistent in part with our results.^34^ *NEAT1* has also been implicated as an epigenetic suppressor of hippocampus-dependent memory and may contribute to age-related memory impairment.^35^ Moreover, *NEAT1* has been reported to be crucial in liver diseases, including non-alcoholic fatty liver disease (NAFLD),^36^ liver fibrosis,^37^ hepatic ischemia/reperfusion injury,^38^ and liver cancer.^39^ *DLEU2* promotes the development of several tumors, including cervical cancer,^40^ non-small-cell lung cancer,^41^ and colorectal cancer.^42^ In HCC, *DLEU2* interacts with enhancer of zeste 2 polycomb repressive complex 2 subunit (EZH2) to promote proliferation, migration, and invasion.^43^ It also promotes HCC cell growth and invasion through the miR*-30a-5p*/*PTP4A1* axis.^44^ Collectively, these observations link both *NEAT1* and *DLEU2* to liver cancer and particularly support a role for *NEAT1* in the regulation of aging and tumor immune microenvironment.

The SASP includes several groups of factors through which senescent cells modify their environment. Accordingly, the effects of senescence in tumors depend strongly on changes in the tumor microenvironment. Chemokines released from senescent cells can act on immune cells, including natural killer (NK) cells and macrophages, that clear senescent cells, limit hepatic stellate-cell proliferation, and thereby prevent liver fibrosis.^45^ In contrast, SASP factors produced by preneoplastic hepatocytes can recruit immature myeloid cells that inhibit NK cells and promote HCC progression.^46^ IL-6 released by stromal cells in the skin-cancer microenvironment can similarly increase immunosuppressive myeloid-derived suppressor cells and inhibit antitumor T-cell responses.^47^ Therefore, we analyzed the influence of the core SASP-associated lncRNA *NEAT1* on immune infiltration in HCC. Our findings suggest that *NEAT1* expression is positively associated with antitumor immunity, and its overexpression indicates favorable prognosis in HCC.

At first glance, the finding that *NEAT1* promotes a proinflammatory SASP appears inconsistent with the better survival of patients whose tumors express high *NEAT1* levels (Fig. 7E). These observations can be reconciled by the context-dependent, opposing effects of the SASP in cancer. In the liver, senescence surveillance is a well-established tumor-suppressive program. In mouse models, senescent premalignant hepatocytes attract CD4^+^ T cells and macrophages, which eliminate the senescent cells and restrict tumor development^48^, ^49^; senescent HSCs are similarly cleared by NK cells.^45^ IL-6 and IL-1β, both positively regulated by *NEAT1* in our experiments, can support T-cell and NK-cell activation rather than suppressing these cells. The TIMER data are consistent with this interpretation: *NEAT1* expression was positively correlated with infiltration by CD8^+^ T cells, CD4^+^ T cells, and macrophages (Fig. 7C and D), which mediate senescence surveillance. In established tumors, the same cytokines can instead promote chronic inflammation and immunosuppression,^13^, ^47^ and the balance between these two SASP functions may shift with disease stage.^46^ Therefore, the survival data suggest that SASP-mediated surveillance predominates in *NEAT1*-high HCC.

Having established a regulatory relationship between *NEAT1* and the SASP, we investigated the underlying molecular mechanism. NF-κB is a principal transcriptional regulator of inflammatory responses and a well-established central regulator of SASP expression.^16^, ^17^, ^18^ Therefore, we hypothesized that *NEAT1* regulates SASP secretion by modulating NF-κB activity. To test this hypothesis, we examined p65 phosphorylation and nuclear translocation in senescent Huh7 cells after *NEAT1* knockdown or pharmacological inhibition of NF-κB. H_2_O_2_-induced senescence significantly increased p-p65 levels and promoted p65 nuclear translocation, indicating robust NF-κB pathway activation. *NEAT1* knockdown markedly attenuated both effects, with inhibition comparable to that produced by the specific NF-κB inhibitor BAY11-7082. Similarly, both *NEAT1* knockdown and BAY11-7082 significantly suppressed the increased secretion of IL-6, IL-8, and IL-1β by senescent HCC cells. These findings indicate that *NEAT1* promotes SASP secretion by activating the NF-κB signaling pathway in senescent HCC cells. To our knowledge, this study is the first to establish a direct mechanistic link between *NEAT1* and NF-κB-mediated SASP regulation in HCC.

## 5 Conclusions

Senescent HCC cells release SASP factors through autocrine and paracrine mechanisms. The cytokines IL-6, IL-8, and IL-1β appear to be positively regulated by lncRNA NEAT1 and negatively regulated by lncRNA DLEU2. NEAT1 may alter the HCC immune microenvironment and inhibit tumor progression by promoting SASP cytokine secretion and recruiting functional immune effector cells. These findings support further investigation of NEAT1 as a potential therapeutic target linking the SASP to the tumor immune microenvironment in HCC.

## CRediT authorship contribution statement

**Yifei Wang:** Writing – original draft, Writing – review & editing. **Runchen Miao:** Writing – original draft, Writing – review & editing. **Sinan Liu:** Formal analysis, Data curation. **Rui Chen:** Formal analysis. **Jie Ren:** Resources. **Kun Yang:** Formal analysis. **Senyi Hu:** Resources. **Kaibo Yang:** Formal analysis. **Kunjin Wu:** Formal analysis. **Xiaohua Ma:** Resources. **Kai Qu:** Supervision, Methodology. **Chang Liu:** Funding acquisition, Data curation. **Ting Lin:** Supervision, Methodology.

## Ethics statement

Not applicable.

## Informed consent

Not applicable.

## Organ donation

Not applicable. This study did not involve organ donation.

## Animal treatment

Not applicable. This study did not involve animal experiments.

## Data availability statement

All data generated or analyzed in this study are available in public databases. The microarray datasets are available from the Gene Expression Omnibus (GEO) under accession numbers GSE19018, GSE19864, GSE36640, and GSE60652. Survival data were obtained from The Cancer Genome Atlas (TCGA).

## Declaration of generative AI and AI-assisted technologies in the writing process

No AI-assisted technologies were used in the writing of this manuscript.

## Funding

This work was supported in part by grants from the 10.13039/501100001809National Natural Science Foundation of China (grant numbers. 82002102 and 81871998); the 10.13039/501100007128Natural Science Foundation of Shaanxi Province (grant numbers 2020JM-373 and 2024JC-ZDXM-49); and the “Basic-Clinical” integrated innovation project of Xi'an Jiaotong University (grant number YXJLRH2022035).

## Declaration of competing interest

The authors declare no conflict of interest. The funders had no role in the design of the study; in the collection, analyses, or interpretation of data; in the writing of the manuscript, or in the decision to publish the results.
